# OA01.03. Sample size and regions of principally achievable significance in cost effectiveness studies: an example of complementary medicine

**DOI:** 10.1186/1472-6882-12-S1-O3

**Published:** 2012-06-12

**Authors:** T Ostermann, K Boehm, F Krummenauer

**Affiliations:** 1 University of Witten/Herdecke, Herdecke, Germany; 2 Institute for Medical Biometrics, University of Witten/Herdecke, Witten, Germany

## Purpose

Health economic studies gain more and more importance in both conventional and complementary medicine. In most cases such studies are conducted as a combination of a RCT and a health economic evaluation (“Piggy-Back-Studies”). Therefore study planning parameters like sample size still are calculated on the basis of the outcome of the clinical parameters. This might lead to situations of underpowering.

## Methods

Based on the sample size estimates given in Glick (2011), we aimed at finding health economic studies of CAM providing mean and standard deviation data on cost and outcome differences. Based on this data and on assumptions on willingness to pay and cost-outcome correlation, we constructed a model of principally achievable significance.

## Results

Based on an existing review on health economic studies in CAM, 8 of 143 studies mentioned sample size calculation parameters. However, only one study (Lin et al. 2008) provided enough data for modelling. We found a significant discrepancy between the sample size of n=46 based on conventional sample size calculation and the hypothetically needed patients of more than 350 based on our modelling approach.

## Conclusion

Planning health economic studies should be done with great caution not to end in a situation of small power. Our approach might retrospectively give estimates of regions of significance and thus might help to interpret health economic studies not only in the field of CAM.

